# Curative Effect of Aqueous Leaf Extract of *Crinum Giganteum* on NMDA-Receptor Antagonist-Induced Schizophrenic Wistar Rat Model

**DOI:** 10.3889/oamjms.2016.061

**Published:** 2016-08-06

**Authors:** Elizabeth Finbarrs-Bello, Emmanuel Nebeuwa Obikili, Esom Emmanuel Anayochukwu, Anyanwu Emeka Godson

**Affiliations:** 1 *Department of Anatomy, College of Medicine, University of Nigeria, UNEC, Enugu, Nigeria*; 2 *Department of Anatomy, Ebonyi State University, Abakaliki Ebonyi State, Nigeria*

**Keywords:** Amygdala, *Crinum giganteum*, Amy Chlorpromazine, Schizophrenia, NMDA, NSE

## Abstract

**AIM:**

This study evaluated the curative potential of *Crinum giganteum* in the treatment of schizophrenia using an NMDA-receptor antagonist-induced schizophrenic Wistar rat model.

**METHODS:**

Twenty-five adult Wistar rats of both sexes of average weights 180 g were divided into two groups: control and schizophrenic rat models. The controls received 0.1 ml of 0. 9% saline, while schizophrenia was induced in models using 25 mg/kg of ketamine hydrochloride (i.p.) for 7 days. On the 8 day models were divided into group’s k1, k2, k3 and k4 of 5 rats each. K1 and the controls were sacrificed then, groups k2 and k3 were treated with 5 mg/kg and 10 mg/kg aqueous leaf extract of *Crinum giganteum* while, k4 (standard) received 25 mg/kg of chlorpromazine orally for 28 days. Amygdala were harvested, processed and stained with Haematoxylin and Eosin (H &E) stain, Neuron-specific enolase (NSE) marker was also used to monitor the curative effect on the amygdala.

**RESULTS:**

Degenerative changes and increased NSE immunoreactivity were observed in the untreated models. Extract-treated models showed normal amygdala and negative NSE immunoreactivity while chlorpromazine treated models revealed decreased NSE immunoreactivity.

**CONCLUSION:**

Crinum giganteum extracts exhibits better curative effect than the standard antipsychotic agent.

## Introduction

In the last three decades, herbal agents have gained popularity and increased patronage due to their folklore use in the management of some disease conditions. The world health organisation (WHO) indices showed that 1% of the world population depend on herbal agents in the treatment of diseases including mental disorders such as schizophrenia [1].

Schizophrenia disturbs thought processes, emotions and regulatory mechanisms involved in the secretion of neurotransmitters: dopamine, serotonin, acetylcholine and glutamate in the limbic areas [2, 3].

The most widely researched are the dopamine hypotheses which implicated diminished secretion of dopamine neurotransmitter in the neurochemistry of schizophrenia [4-8]. However, the glutamate hypothesis of schizophrenia posits dysfunction of the N-methyl-D-aspartate (NMDA) glutamate receptor in schizophrenia. The NMDA receptors are a major subtype of glutamate receptors and mediate slow excitatory postsynaptic potentials (EPSPs). These slow EPSPs are considered critical for the proper expression of complex behaviours, such as associative learning, working memory, behavioural flexibility and attention which are impaired in schizophrenia [4, 5]. In early development, it aids the development of neural pathways whose malfunction may lead to susceptibility to schizophrenia [1].

Evidence from basic and clinical researchers show that genes associated with the risk for schizophrenia influence the modulatory sites on the NMDA receptor or intracellular receptor interacting proteins that link glutamate receptors to signal transduction pathways [9]. A postmortem study reported changes in glutamate receptor binding, transcription, and subunit protein expression in the prefrontal cortex, thalamus, and hippocampus of subjects with schizophrenia [10]. Antagonists of the NMDA receptor elicit schizophrenic symptoms in recreational use or administration of a single low dose of such agents [11, 12]

Phencyclidine (PCP) or ketamine produces “schizophrenia-like” symptoms that resemble positive (delusion and hallucination), negative (avolition, apathy, and blunted affect), and cognitive deficits in healthy individuals and rodents [4, 5, 13-16]. In addition, the NMDA glutamate receptor regulates the function of other neurotransmitter systems implicated in the pathophysiology of schizophrenia [17].

In pharmacotherapy, dopamine transmitter is the target of most antipsychotic drugs for schizophrenia and NMDA receptor antagonist-induced schizophrenia [7]. Generally, antipsychotics are able to manage symptoms like delusion, hallucination and aggression [18-20]. They are still best described as control measures as they do not totally cure the mental disorders. Thence, agents that modulate glutamate via the NMDA receptors promise to be a treatment entity towards the discovery of better pharmacological target and agents that could treat schizophrenia besides dopamine.

*Crinum giganteum* is a major herb used in the treatment of mental illnesses in some parts of Africa like Cameroun, Niger Republic and Nigeria. In Nigeria, it is predominantly used in northern where it called gadalli, Albacce Buru or Albacce Dawaddi [21]. Traditional medicine practitioners in the region have claimed that *Crinum giganteum* could cure schizophrenia and other mental condition [22]. It’s been known that herbal agents could have a toxic effect, lacks standard formulation and adequate dosing regimen [22]. To authenticate this claim, scientific evaluation of the plant is necessary; this study evaluates the curative potential of aqueous leaf extract of *Crinum giganteum* on the amygdala using a NMDA receptor antagonist to induce schizophrenia system in Wistar rats.

This was aimed at ascertaining the curative effect using neuron-specific enolase (NSE) marker and comparing this property with a standard antipsychotic (chlorpromazine) agent.

## Materials and Methods

### Collection and authentication of plant materials

The leaves were procured from the open market and identified by the curator Prof. Mrs M.O Nwosu of the Department of Plant Science and Biotechnology, University of Nigeria, Nsukka as *Crinum giganteum*. Herbarium sheet was prepared and a voucher specimen (UNH/13/401) was deposited at the herbarium of same Department.

### Preparation of plant extract

The leaves were washed with distilled water and air-dried under shade for seven days. Thereafter, the leaves were pulverised into a fine powder, 100 grammes of the dried leaf powder was placed in a beaker containing 500ml of distilled water. The mixture was heated on a hot plate with continuous stirring at 30 ºC -40ºC for 20 minutes. It was then allowed to cool and then filtered through mesh cloth. The filtrate (aqueous extract) was evaporated to a paste using a vacuum evaporator. This was transferred into a suitable container and kept in the refrigerator at low temperature (4 ºC) for the experiment.

### Animals and ethical concern

Twenty-five (25) adult Wistar rats of both sexes of average weights 180 g were purchased from the animal house of the College of Medicine. The university of Nigeria, and housed at the animal facility of the same college. The animals were housed in netted iron cages in groups of five, fed with grower’s mash and provided water *ad libitum*. The rats were maintained under laboratory conditions (temperature 24±2 °C with relative humidity 60-70%, and 12-hour light-dark cycle). They were acclimatised for two weeks before the experiment. The experimental protocols and techniques used in the study were in accordance with accepted principles for laboratory animal use and care. The study was reviewed and approved by the University Health Research Ethics Committee with certificate number NKREC/05/01/2008B-FWA00002458-1RB00002323.

### Induction of schizophrenia in rat models

Twenty (20) rats were induced using 25 mg/kg ketamine hydrochloride (a NMDA receptor antagonist) per body weight, intraperitoneal (i.p), for 7 days. The control (group A) had 5 rats which received 0.1ml 0.9% saline. The animals exhibited side to side head rocking and continuous staggering locomotion.

### Treatment of animals and tissue processing

On the 8 days, the ketamine group (n = 20) was divided into four groups (k1-k4). The control (group A) and group k1 (untreated model) were sacrificed same day. Groups k2 and k3 received 5 mg/kg and 10 mg/kg of aqueous leaf extract of gadalli orally respectively, while group k4 received 25 mg/kg of chlorpromazine orally for 28 days. The rats were anaesthetized with 50 mg/kg thiopental sodium and aortic perfusion fixation with 4% paraformaldehyde was carried out. The brains were dissected out and further fixed in 4% paraformaldehyde overnight, amygdala was harvested. Fixed tissues were dehydrated in ascending grades of ethanol (50%, 70%, 90% and 100%), cleared in xylene and embedded in paraffin wax. Serial sections of 10µm thick were obtained using a rotatory microtome. Part of the paraffinized sections was stained using haematoxylin and eosin (H &E) and the rest were used for the immunohistochemical study.

### Immunohistochemical demonstration of NSE

The Avidin-Biotin Complex (ABC) method also referred to the Avidin-biotin Immunoperoxidase method was used. Paraffin processed tissues were sectioned at 2 microns on the rotary microtome and placed on the hot plate at 70 degrees for at least 1hour. Sections were brought down to water by passing the on 2 changes of xylene, then 3 changes of descending grades of alcohol (100%, 90%, 70% and 50%) and finally to water. Antigen retrieval was performed on the sections by heating them in a citric acid solution of PH 6.0 using the microwave at power 100v for 15 minutes. The sections were equilibrated gradually with cool water to displace the hot citric acid for at least 5 minutes for the section to cool. Peroxidase blocking was done on the sections by simply covering sections with 3% hydrogen peroxide for 15 mins. Sections were then washed with phosphate-buffered saline (PBS) and protein blocking was performed using avidin for 15 mins. Sections were washed again with PBS and endogenous biotin in tissues was blocked using biotin for 15 mins.

After washing with PBS sections were incubated with the respective diluted primary antibody NSE antibody (diluted 1:100) for 60 mins. Excess antibodies were washed off with PBS and a secondary antibody (LINK) was applied on the section for 15 min. Sections were washed and the (LABEL) which is the horseradish peroxidase (HRP) was applied on all sections for 15mins. A working DAB (3, 3’-diaminobenzidine) solution was made up by mixing 1 drop (20 microns) of the DAB chromogen to 1ml of the DAB substrate. This working solution was applied on sections after washing off the HRP with PBS for at least 5mins. The brown reactions begin to appear at this moment especially for positive targets. Excess DAB solution and precipitate were washed off with water. Sections were counterstained with Haematoxylin solution for at least 2 mins and blued briefly. Sections were dehydrated in alcohol, cleared in xylene and mounted in DPX.

## Results

**Figure 1:**
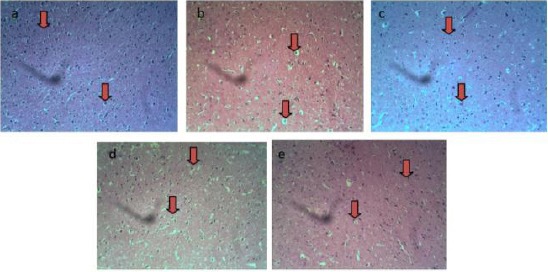
Sections of amygdala of rats (a) control (0.1ml saline), (b) untreated schizophrenic model (25 mg/kg ketamine), (c) and (d) schizophrenic models treated with 5 mg/kg and 10 mg/kg of ethanolic leaf extract of Crinum giganteum, and (e) schizophrenic model treated with 25 mg/kg of chlorpromazine. Arrows. (H&E) x 200

We can see from the Figure 1 that sections of amygdala of rats (a) control (0.1ml saline) shows normal neurons, (b) untreated schizophrenic model (25 mg/kg ketamine) shows cytoplasmic vacuolations, (c) and (d) schizophrenic models treated with 5 mg/kg and 10 mg/kg of ethanolic leaf extract of *Crinum giganteum* shows normal neurons respectively, and (e) schizophrenic model treated with 25 mg/kg of chlorpromazine shows relatively normal neuron.

**Figure 2:**
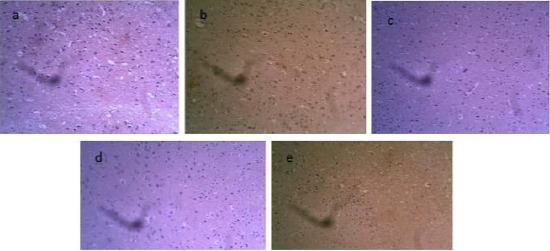
Sections of amygdala of rats (a) control (0.1ml saline), (b) untreated schizophrenic model (25 mg/kg ketamine), (c) and (d) schizophrenic models treated with 5 mg/kg and 10 mg/kg of ethanolic leaf extract of Crinum giganteum, and (e) schizophrenic model treated with 25 mg/kg of chlorpromazine. NSE x 200

Sections of amygdala of rats (a) control (0.1ml saline) show negative immunoreactivity, (b) untreated schizophrenic model (25 mg/kg ketamine) shows positive NSE immunoreactivity, (c) and (d) schizophrenic models treated with 5 mg/kg and 10 mg/kg of ethanolic leaf extract of *Crinum giganteum* shows NSE negative immunoreactivity respectively, and (e) schizophrenic model treated with 25 mg/kg of chlorpromazine shows positive NSE immunoreactivity (Figure 2).

## Discussion

In this study, ketamine-induced neuronal damage was characterised by cytoplasmic vacuolation and eccentric nuclei. Ketamine induction of vacuolation and neuronal cell death in rodents has been established [7, 23, 24]. This is possible considering the mechanisms propounded from previous studies via inhibition or antagonism of N-Methyl-D- Aspartate (NMDA) receptors [23, 24].

Compensatory upregulation of NMDA receptor expression, which is tied to the toxic influx of calcium and elevated reactive oxygen species (ROS) generation and neuronal cell death [25, 26]. Another mechanism involves blockade of an excitatory NMDA glutamate receptor on the GABA neurones, which could trigger decrease GABA release and activate compensatory increase blood flow and metabolism [27]. Ketamine neurotoxicity also triggers induction of heat shock proteins (Hsp70) and denaturation of intracellular proteins in pyramidal neurones [27-29]

The neuronal damage (ketamine neurotoxicity) observed in the untreated schizophrenic models was attenuated by treatment with the varying doses of the extract of *crinum giganteum*. Meanwhile, the standard antipsychotic treatment showed relatively normal neurone which was less prominent compared to the extract treatment. Their potential or degree of the effects can further be deduced from the NSE immunoreactivities.

Neuron-specific enolase (NSE) is expressed in all neuronal cell types, its detection has been used to identify neuronal cells and monitor disease progress in the CNS [30]. The untreated schizophrenic rat models showed positive and increased expression of NSE which confirms the neuronal damage earlier reported in the group. This was consistent with increased NSE levels seen in acute neuronal injury in the area of the brain [31]. The negative NSE immunoreactivity in the extract treated groups and the verisimilitude with the control attest to that fact that ketamine effect was reversed and the amygdala integrity was restored. However, curative effect of the standard antipsychotic treatment (chlorpromazine) was less compared to the extract treatment going by the positive but decreased NSE immunoreactivity.

We attributed the effect of the extract to the phytochemicals present in the aqueous leaf extract of *crinum giganteum* such as alkaloids, saponins and glycosides. Saponins generally exhibit antioxidant activity and ginsenosides saponins are known to foster neurogenesis [23, 32]. Similarly, glycosides possess neuroprotective effect [33, 34], which must have played a role in the reversal effect of the extract. The activities of these phytochemicals in the extract have conferred neuroprotective effects by attenuating effect of the NMDA receptor antagonist (ketamine) in the treated schizophrenic rat models.
